# Long non-coding RNA FAM83H-AS1 acts as a potential oncogenic driver in human ovarian cancer

**DOI:** 10.1186/s13048-020-00756-y

**Published:** 2021-01-07

**Authors:** Xiaolei Yuan, Ying Huang, Man Guo, Xiaowei Hu, Peiling Li

**Affiliations:** 1 Department of Obstetrics and Gynecology, The Second Affiliated Hospital of Harbin Medical University, 246 Xuefu Road, Nangang District, Harbin, 150081 Hei Longjiang Province China; 2 Harbin Medical University, 157 Baojian Road, Nangang District, Harbin, 150081 Hei Longjiang Province China; 3 Medical ward 7, Cancer Hospital Affiliated to Harbin Medical University, 150 Haping Road, Nangang District, Harbin, 150081 Hei Longjiang Province China

**Keywords:** Ovarian cancer, lncRNA FAM83H-AS1, Network, Survival, Immune

## Abstract

**Objective:**

Ovarian cancer (OC) is one of the most aggressive women cancers with increasing incidence and mortality rates worldwide. Long non-coding RNAs (lncRNAs) could as major players in OC process. Although FAM83H antisense RNA1 (FAM83H-AS1) is demonstrated play an important roles in a many cancers, the detailed function and mechanism has not been reported in OC.

**Results:**

We integrated multiple kinds of bioinformatics approaches and experiments validated method to evaluate functions of FAM83H-AS1 in OC. Some differential expressed lncRNAs were identified between OC and normal control tissues. FAM83H-AS1 was one of most differentially expressed lncRNAs and up-regulated in multiple cancer types. Specially, expression of FAM83H-AS1 was higher in OC and showed difference in diverse stages. High FAM83H-AS1 expression is associated with worse pan-cancer and OC outcomes. FAM83H-AS1-centric network including lncRNA-miRNA, lncRNA-protein and lncRNA-mRNA ceRNA network were constructed to infer the function and mechanism of FAM83H-AS1. There were two methylation sites including cg01399317 and cg20519035 located at FAM83H-AS1. The methylation level of cg01399317 was correlated with gene expression of FAM83H-AS1. The expression level of FAM83H-AS1 was correlated with infiltration level of immune cell including macrophage, neutrphil and dendritic cell in OC patients. Lastly, qRT-PCR showed that the expression of FAM83H-AS1 was higher in OC tissues than normal control tissues.

**Conclusion:**

Collectively, these results indicated that FAM83H-AS1 may act as an oncogenic driver and it may be a potential therapy target in OC.

## Background

In the female reproductive system, ovarian cancer (OC) is one of the most malignant tumors [1]. As cancer statistics in China and United States suggested that the mortality rate of OC has been rising for the past few years [2, 3]. there will be approximately 22,240 new cases of ovarian cancer diagnosed and 14,070 ovarian cancer deaths in the United States [4]. OC accounts for 2.5% of all female malignant tumors and part of deaths for cancer patients due to poor survival which largely driven by late stage diagnoses [5]. Surgical resection combined with platinum-based and taxane-based chemotherapy is the major and standard approach for advanced-stage ovarian treatment [6]. Although advancing insight about mechanism and treatment of OC has been evolved rapidly in recent year, survival rates have improved only slightly over the past 3 decades. Thus, Improving prevention and early detection based on identifying molecular biomarkers are effective ways to enhance survival for OC patients.

Long non-coding RNAs (lncRNAs), which are defined as RNA transcripts of > 200 nucleotides that are not translated into protein [7]. lncRNAs are a highly versatile class of transcripts that have sparked new lines of research in nearly all fields of the life sciences [8]. More recently, emerging studies have identified lncRNAs as major players in many kinds of cancer processes [9]. Many studies revealed that lncRNA play essential roles in proliferation, migration, and invasion of cancers including OC [10, 11]. For example, lncRNA LINC00176 was highly expressed in OC tissues as well as in OC cell lines, respectively. Knockdown of lncRNA LINC00176 suppresses OC progression by BCL-mediated down-regulation of ceruloplasmin [12]. Li et al. suggested that lncRNA UCA1 was upregulated in cisplatin-resistant patient tissues and cell lines. Knockdown of UCA1 inhibited cell proliferation and promoted the cisplatin-induced cell apoptosis in OC cells [13]. The expression of lncRNA ABHD11-AS1 in OC tissues was higher compared to normal ovarian tissue. Overexpression of ABHD11-AS1 promoted OC cell proliferation, invasion and migration, and inhibited apoptosis [14]. Specially, Emerging evidence indicates that lncRNAs participate in crosstalk between tumor and tumor immune microenvironment [15]. The regulatory mechanisms of lncRNAs were multiple and complex such as competing endogenous RNA (ceRNA) in cancer including OC [16].

Recently, a novel lncRNA, FAM83H antisense RNA1 (FAM83H-AS1), demonstrated important roles in a many cancers. Zhang et al. reported that FAM83H-AS1 is associated with clinical progression and modulates cell proliferation, migration, and invasion in bladder cancer [17]. FAM83H-AS1 was found overexpressed in HPV-16 positive cervical cancer cell lines in an HPV-16 E6-dependent manner but independently of p53 regulation [18]. Xu et al. indicated that the expression of FAM83H-AS1 was higher in glioma tissues and cell lines and overexpression of FAM83H-AS1 was associated with poor prognosis of glioma. Specially, FAM83H-AS1 was upregulated in OC [19]. However, underlying mechanisms of FAM83H-AS1 regulating functions in OC have yet to be elucidated.

In present study, differential expressed lncRNAs were identified between OC and normal ovarian tissues. FAM83H-AS1 was differential expressed in many types of cancers. Specially, the expression of FAM83H-AS1 was higher in OC and fluctuated among diverse stage of OC. FAM83H-AS1 was also associated with survival in pan-cancer and OC. FAM83H-AS1-centric network including lncRNA-miRNA, lncRNA-protein and lncRNA-mRNA ceRNA network were constructed. qRT-PCR showed that FAM83H-AS1 was up-regulated in OC tissues. Overall, our findings indicated that FAM83H-AS1 may mediate the oncogenesis process and can be regarded as a prognostic biomarker in OC.

## Results

### Some dysregulated genes and lncRNAs were identified in OC

**Fig. 1:**
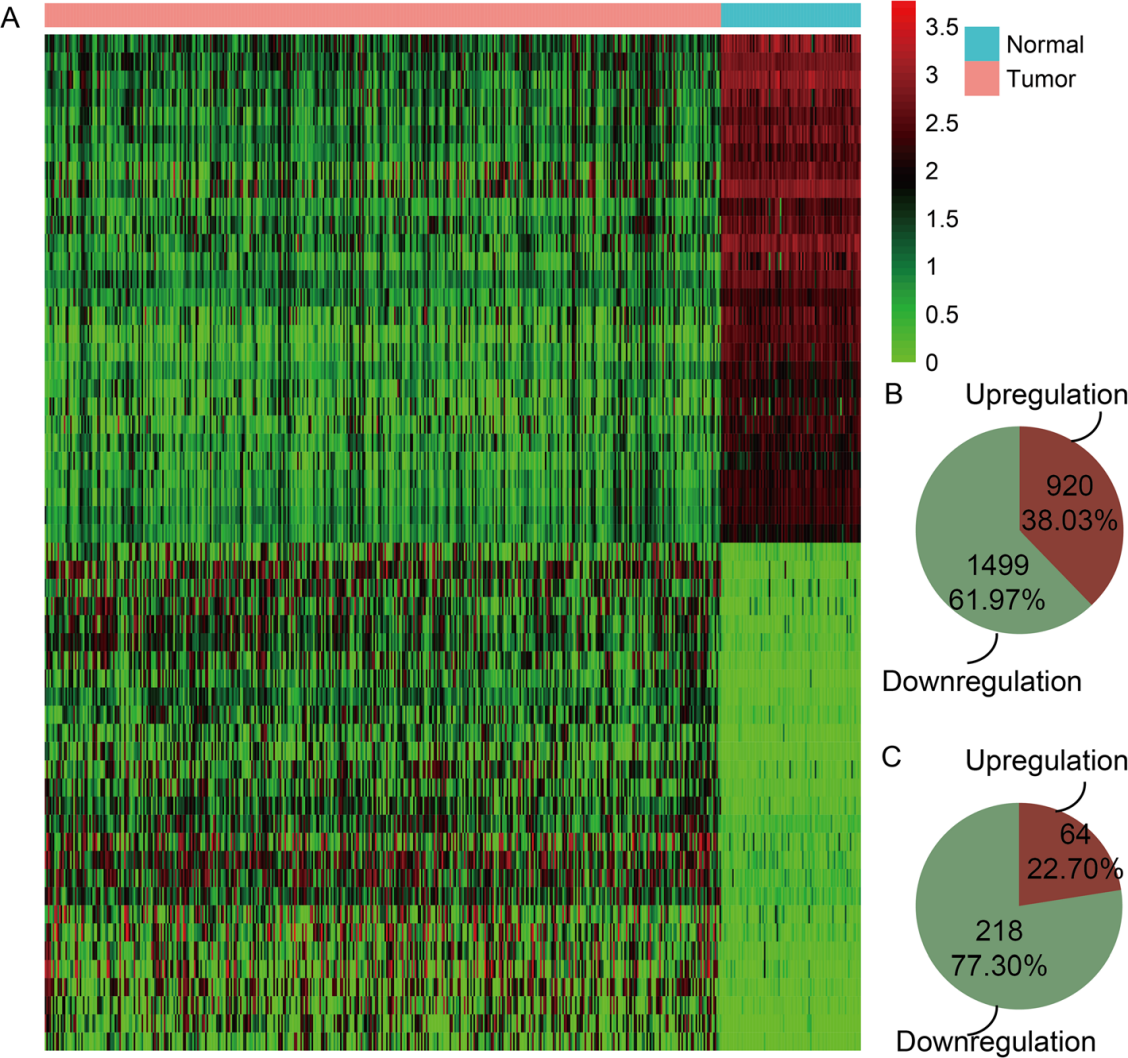
Some dysregulated genes and lncRNAs were identified in OC. **a** Heatmap of significantly differentially expressed lncRNAs and coding genes in OC patients. Red indicates genes that had higher expression level and green indicates genes had lower expression. **b** The pie chart shows percent of up- and down-regulated lncRNAs and coding genes. **c** The pie chart shows percent of up- and down-regulated lncRNAs

We identified differential expressed coding genes and lncRNAs between OV tissues and normal control tissues to screen candidate genes. 2419 differential expressed lncRNAs and coding genes were identified based on t-test (*P* < 0.001) for OV patients (Fig. 1a). There were 920 (38.03%) up-regulated and 1499 (61.97%) down-regulated for all the differential expressed lncRNAs and coding genes (Fig. 1b). We further focused on dysregulated lncRNAs in OC. There were 64 (22.70%) and 218 (77.30%) up and down-regulated lncRNAs in OC tissues compared with normal ovarian tissues (Fig. 1c). The results indicated that some dysregulated lncRNAs maybe play essential roles in OC patients. Specially, differential expression of lncRNA FAM83H-AS1 was extremely significant (*P* = 1.72E-101, fold change value = 3.781). Thus lncRNA FAM83H-AS1 was considered as a candidate biomarker for OC to perform follow analyses.

### FAM83H-AS1 expression is dysregulated in human pan-cancer and OC

**Fig. 2:**
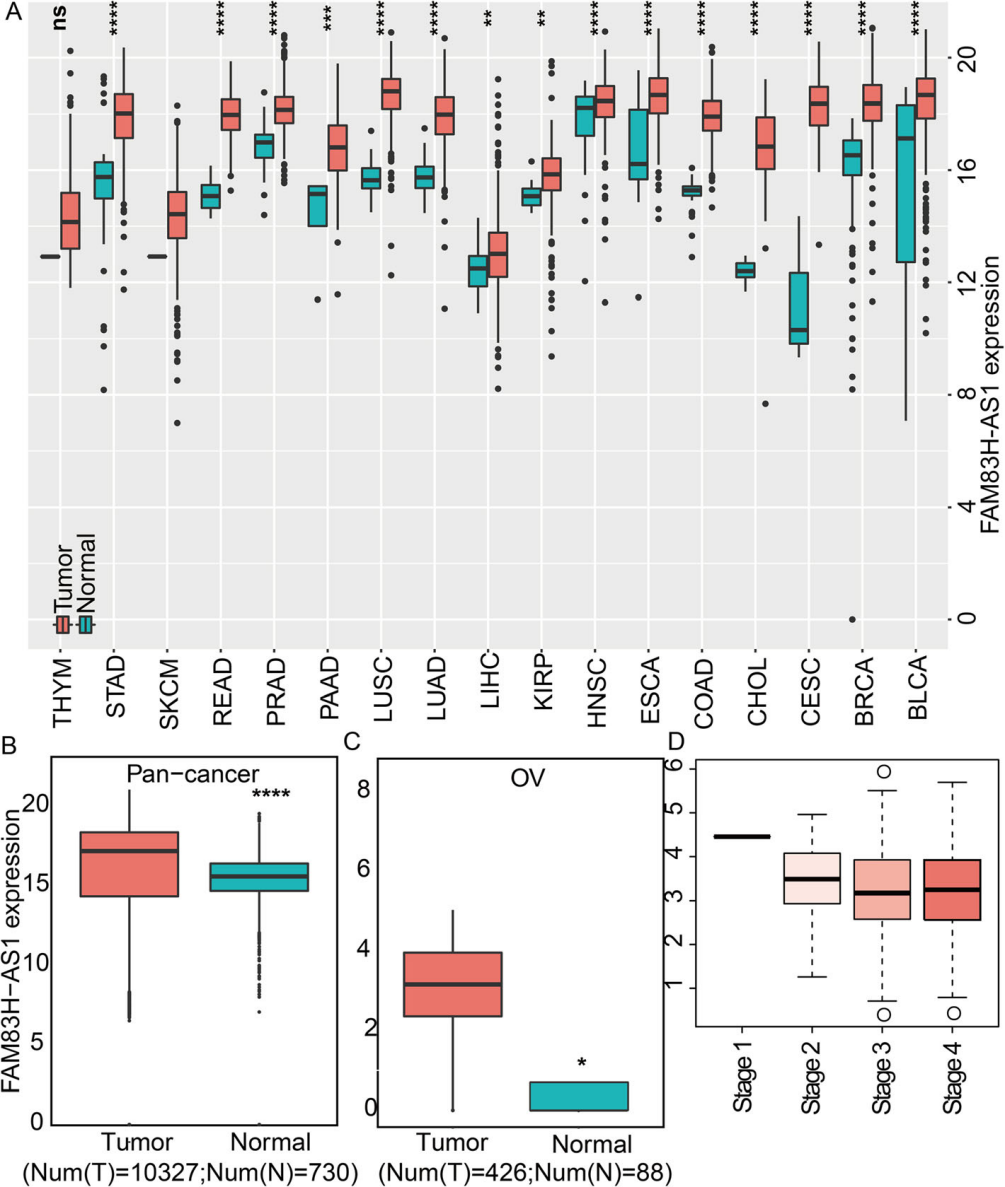
FAM83H-AS1 expression is upregulated in human pan-cancer and OC. **a** The box plots shows the expression of FAM84H-AS1 in multiple cancer types. Red and blue represent tumor and normal samples. **b** The expression of FAM84H-AS1 in pan-cancer. **c** The expression of FAM84H-AS1 in OC. **d** The expression of FAM84H-AS1 in diverse stages of OC

In order to characterize FAM83H-AS1 in OC, we first explore the expression of FAM83H-AS1 other cancers. We downloaded and analyzed TCGA data to assess the expression of FAM83H-AS1 in pan-cancer. The result indicated that expression of FER1L4 in all the cancer types including bladder urothelial carcinoma (BLCA); breast invasive carcinoma (BRCA); cervical squamous cell carcinoma (CESC); cholangiocarcinoma (CHOL); colon adenocarcinoma (COAD); esophageal carcinoma (ESCA); head and neck squamous cell carcinoma (HNSC); lung squamous cell carcinoma (LUSC); liver hepatocellular carcinoma (LIHC); lung adenocarcinoma (LUAD); kidney renal papillary cell carcinoma (KIRP); prostate adenocarcinoma (PRAD); rectum adenocarcinoma (READ); skin cutaneous melanoma (SKCM); stomach adenocarcinoma (STAD); pancreatic adenocarcinoma (PAAD); thymoma, (THYM) and ovarian serous cystadenocarcinoma (OV) was significantly differential compared with their noncancer counterparts (Fig. 2a). And the expression of FAM83H-AS1 was up-regulated in any cancer type. FAM83H-AS1 was also dysregulated in integrated pan-cancer patients (Fig. 2b). The abnormal expression of FAM83H-AS1 was also observed in OC (Fig. 2c). In addition, we also found that expression level of FAM83H-AS1 changed in diverse stages of OC (Fig. 2d).

### FAM83H-AS1 was associated with prognosis in pan-cancer and OC

**Fig. 3:**
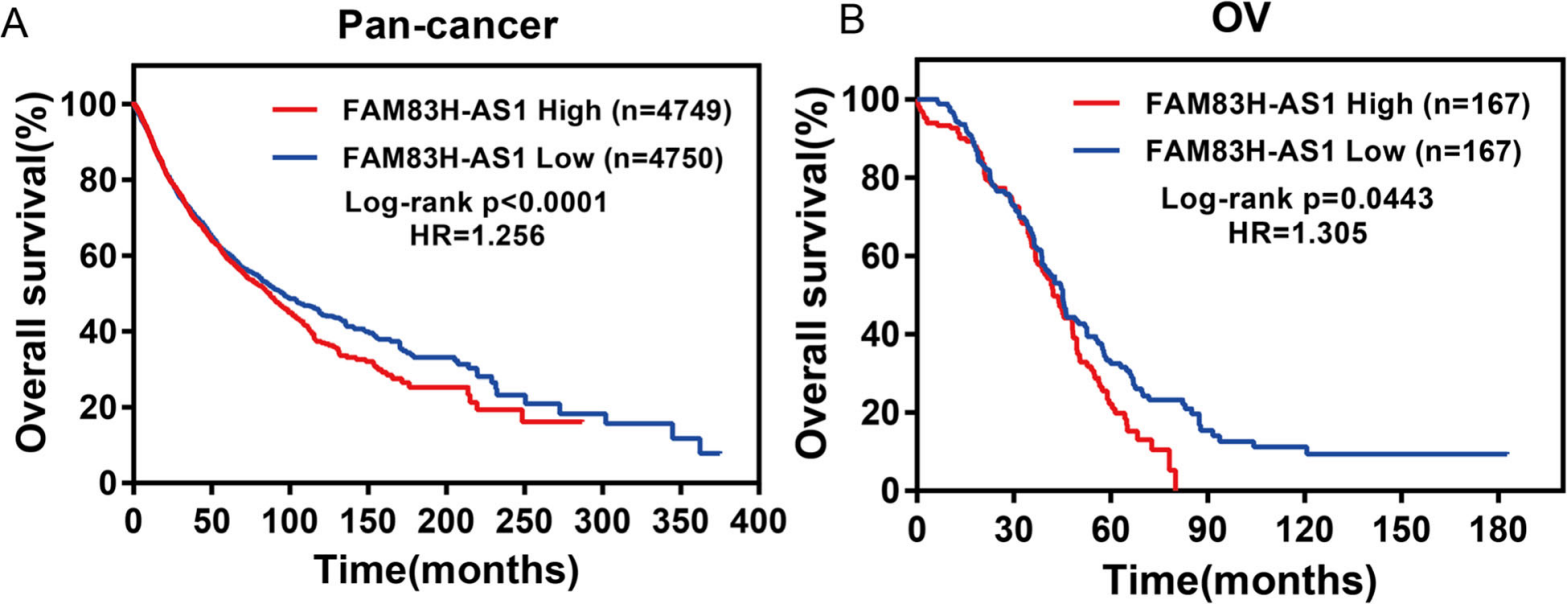
Kaplan-Meier survival curves for FAM83H-AS1 associated with overall survival. **a** High expression of FAM83H-AS1 correlates with worse OS in pan-cancer patients and (**b**) OC patients. X- and Y-axis represent survival time and overall survival. Red and blue lines represent high and low expression of FAM83H-AS1

Kaplan–Meier analysis and the log-rank test were used to explore the association between FAM83H-AS1 expression and survival status in OC patients in present study. We found higher expression of FAM83H-AS1 was associated with worse overall survival in pan-cancer (*P* < 0.0001, Fig. 3a). As except, higher expression of FAM83H-AS1 was also associated with worse overall survival in OC patients (*P* = 0.044, Fig. 3b). The results indicated that lncRNA FAM83H-AS1 maybe could as a unfavorable prognostic biomarker for OC patients.

### FAM83H-AS1-centric lncRNA-miRNA, lncRNA-protein and lncRNA-mRNA ceRNA networks could show the special function of FAM83H-AS1

**Fig. 4:**
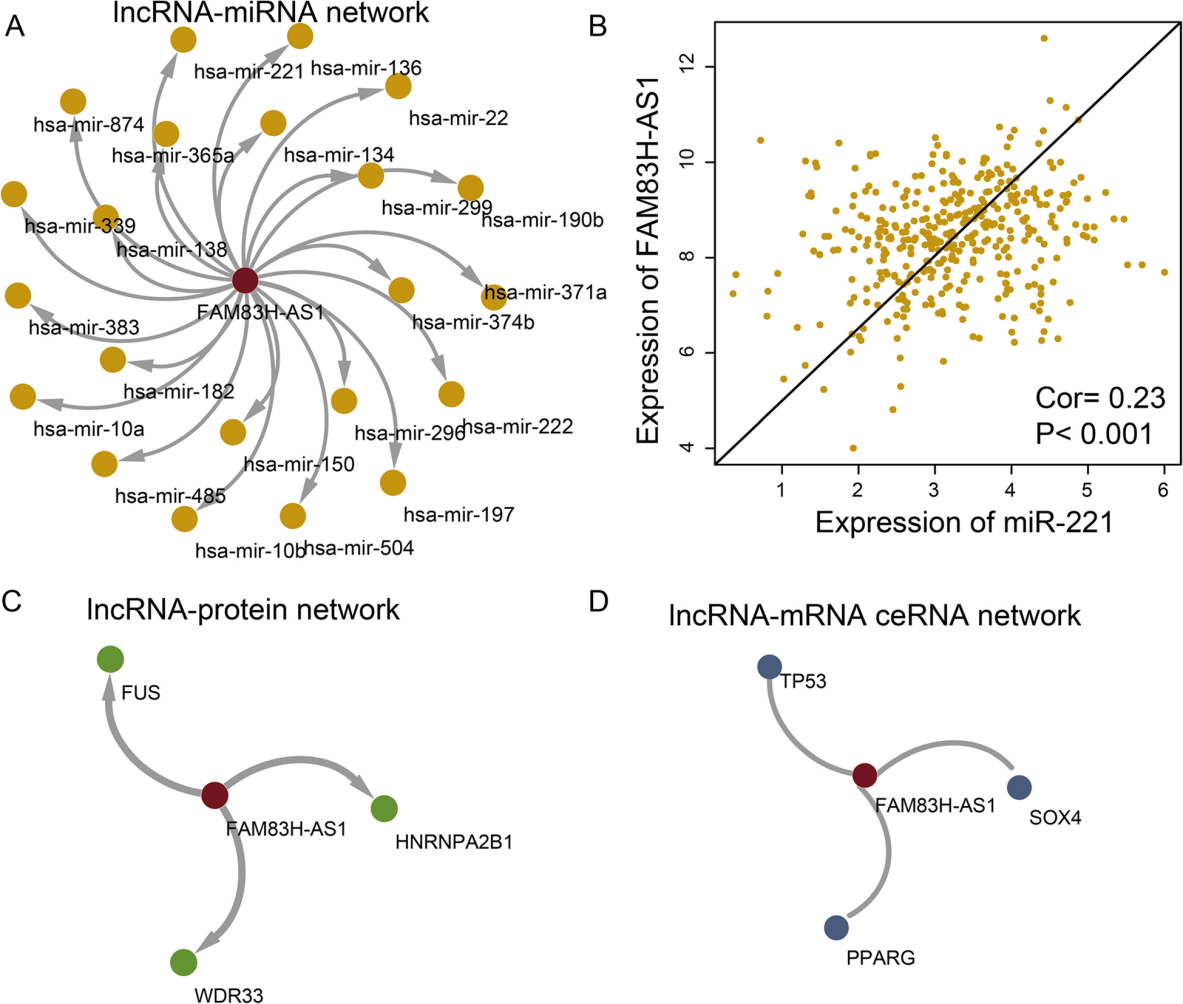
FAM83H-AS1-centric lncRNA-miRNA, lncRNA-protein and lncRNA-mRNA ceRNA networks. **a** FAM83H-AS1-centric lncRNA-miRNA network. Red and yellow represent lncRNAs and miRNAs. **b** The point plot shows expression of miR-211 (X-axis) and FAM83H-AS1 (Y-axis) in OC. **c** FAM83H-AS1-centric lncRNA-protein network. Red and green represent lncRNAs and proteins. **d** FAM83H-AS1-centric lncRNA-mRNA ceRNA networks. Red and blue represent lncRNAs and mRNAs

We try to explore the function of FAM83H-AS1 based on studying its interacted miRNA and coding genes using multiple networks. First, a lncRNA-mRNA network was constructed, which contained 22 interacted miRNAs (Fig. 4a). The co-expression of FAM83H-AS1 and any interacted miRNA were also calculated. The expression of miR-211 was significantly positive correlated with FAM83H-AS1 in OC patients (Fig. 4b). Present study demonstrated that the expression of miR-211 is differential expressed in OC tissues and cell lines compared to normal epithelial ovarian tissue and human ovarian surface epithelial cells, respectively. miR-211 was also found to arrest cells in the G0/G1-phase, inhibit proliferation and induce apoptosis [20]. Second, a lncRNA-protein (or coding gene) network was constructed (Fig. 4c). There were three coding genes could interacted with FAM83H-AS1. These three coding genes all play essential roles in cancers [21–23]. At last, we also constructed a lncRNA-mRNA ceRNA network (Fig. 4d). TP53 was a ceRNA of FAM83H-AS1 and mutations in the TP53 gene are still by far the most frequent genomic event in cancer genomes [24]. All the results indicated that FAM83H-AS1 maybe play its role by interacting with some OC-related coding genes and miRNAs.

### Expression level of FAM83H-AS1 was associated with methylation and immune

**Fig. 5:**
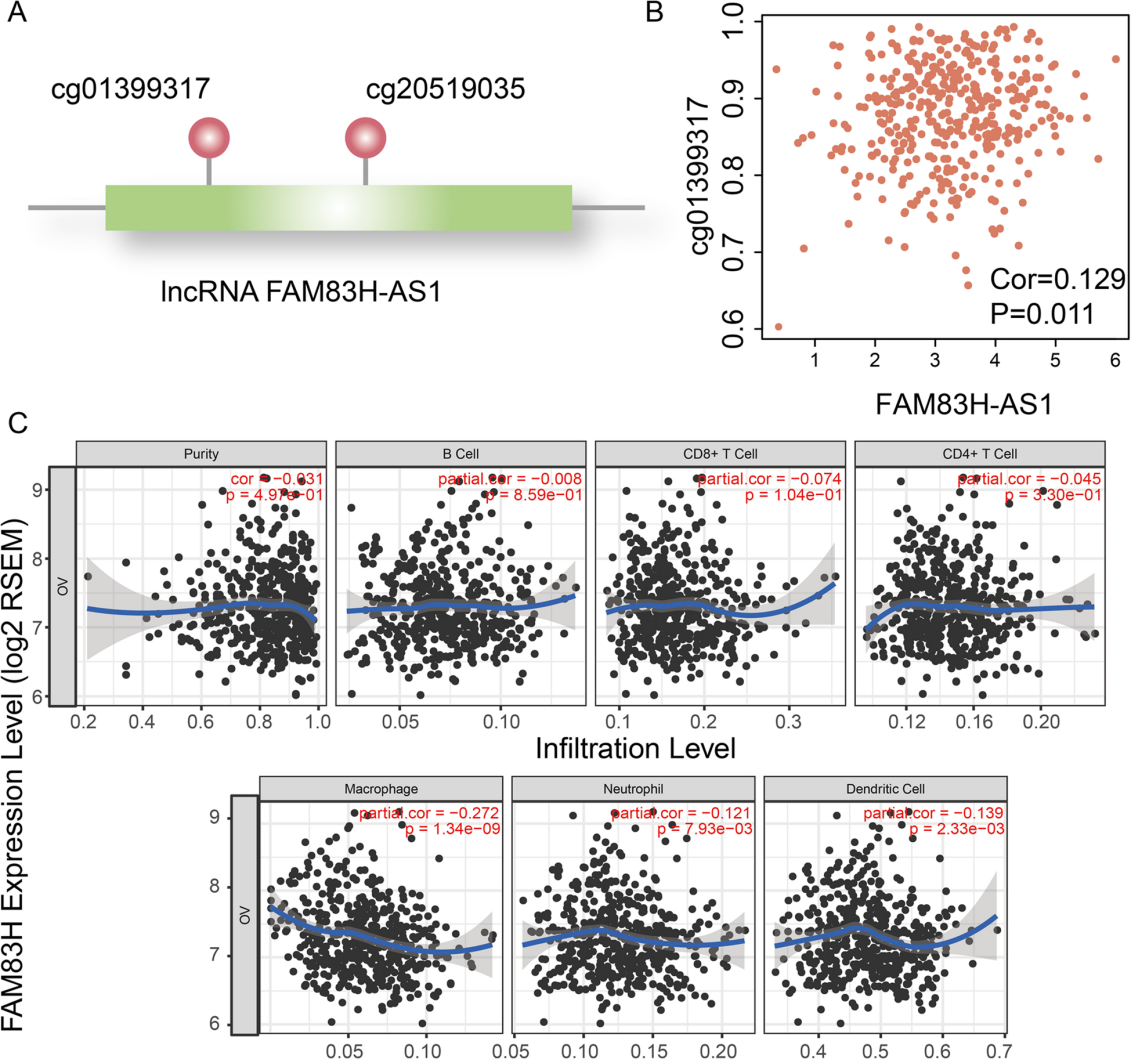
Expression level of FAM83H-AS1 was associated with methylation and immune. **a** There are two methylation sites locate at FAM83H-AS1. **b** The point plot shows expression of FAM83H-AS1 (X-axis) and methylation level of cg01399317 (Y-axis) in OC. **c** The point plots show the correlations between FAM83H-AS1 and immune cells including B cell, CD8+ T cell, CD4+ cell, Macrophage, Neutrophil and Dendritic cell. X- and Y-axis represent infiltration level and FAM83H-AS1 expression

We found there were two methylation sites including cg01399317 and cg20519035 located at FAM83H-AS1 (Fig. 5a). The methylation level of cg01399317 was correlated with gene expression of FAM83H-AS1 (*P* = 0.011, Fig. 5b). Recent years, immunotherapy have resulted in clinical success in treating late-stage cancers [25]. Some immune signature genes have also been identified and could be applied to characterize immune infiltrates and predict clinical outcome for cancers. We also explored the associations between immune and FAM83H-AS1. The expression level of FAM83H-AS1 was associated with infiltration level of immune cell including macrophage (*P* = 1.34e-9), neutrphil (*P* = 7.93e-03) and dendritic cell (*P* = 2.33e-03) in OC patients (Fig. 5c). The results indicated that FAM83H-AS1 maybe could be used to characterize immune infiltrates for OC patients.

### FAM83H-AS1 was up-regulated in OC tissues by qRT-PCR validation

**Fig. 6:**
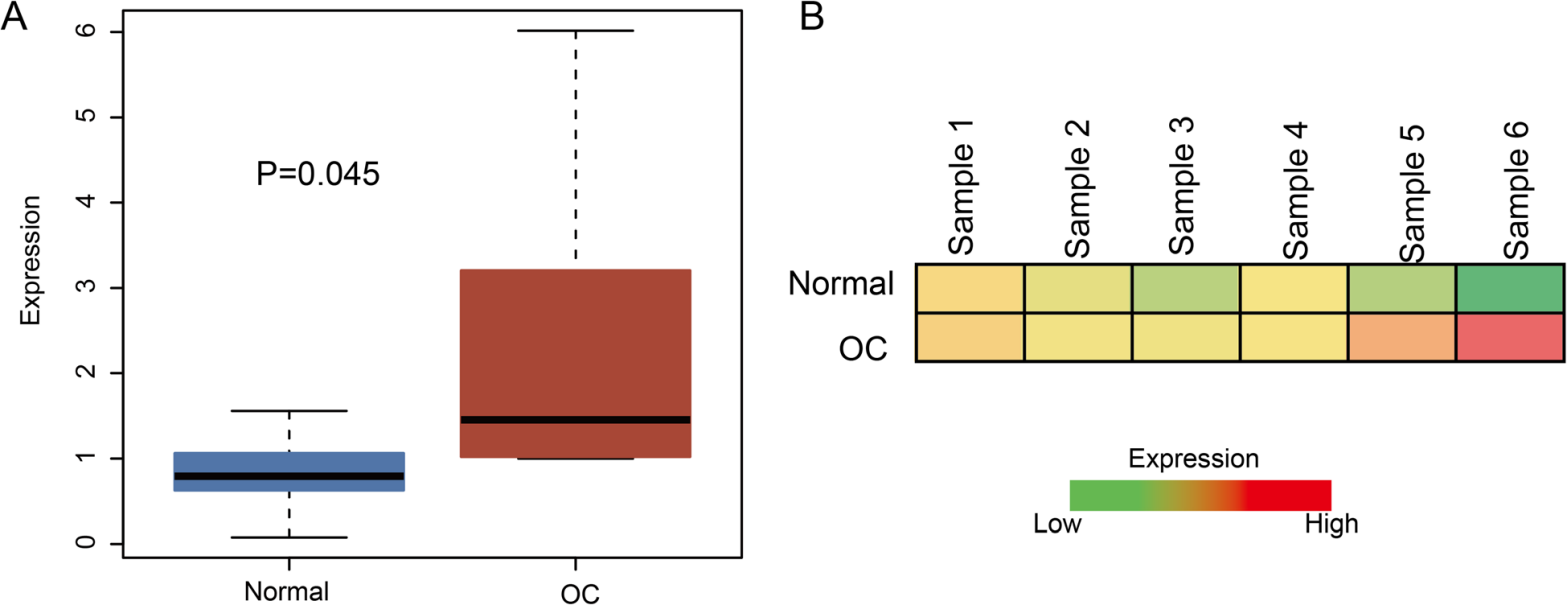
Expression levels of FAM83H-AS1 in OC by qRT-PCR. **a** Relative expression levels of FAM83H-AS1 in OC tissue and adjacent noncancerous tissues. **b** The heatmap shows the expression of FAM83H-AS1. Red and green represent high and low expression level

FAM83H-AS1 levels was significantly differentially expressed in OC tissues compared with corresponding adjacent non-tumor tissues (*P* = 0.045, Fig. 6a). The fold-change value of FAM83H-AS1 was 2.9 in OC and control tissues. The expression level of FAM83H-AS1 was significantly higher in OC tissues than control tissues (Fig. 6b).

## Discussion

Multiple lines of evidence suggested that non-coding RNAs are majority of genome transcripts, however, only a small part of them have been characterized to be biologically functional [26]. In recent years, more and more studies focused on implicating the role of lncRNAs in diseases including cancers [27–29]. Some studies had reproted that lncRNA FAM83H-AS1 was associated with many types of cancers such as breast cancer [30], colorectal cancer [31] and colon cancer [32]. However, the function and mechanism of FAM83H-AS1 in OC has not been systemically studied.

In present study, we found FAM83H-AS1 as a novel and essential lncRNA involved in OC. We showed that FAM83H-AS1 was significantly upregulated in many kinds of cancer tissues compared with control normal tissues. In OC patients, FAM83H-AS1 was one of top differential expressed lncRNAs. Specially, expression of FAM83H-AS1 also showed difference in diverse stage of OC. Higher expression of FAM83H-AS1 was associated with worse overall survival in pan-cancer and OC. All above results indicated that FAM83H-AS1 may act as an oncogenic driver in OC.

In order to further explore the biological function and mechanism of FAM83H-AS1, some regulatory networks were constructed. Previous studies suggested that interacted or co-expressed coding genes could represented the function of lncRNAs [33]. Thus, we try to use the function of coding genes and miRNAs to estimate the role of FAM83H-AS1 in OC. In present study, the expression of miR-211 was significantly positive correlated with FAM83H-AS1 in OC patients. The role of miR-211 in diagnosis, prognosis and treatment in OC had been reported in many studies. For example, wang et al. found that miR-211 inhibited most of DNA damage response-related genes, and proposed that miR-211 might affect the sensitivity of OC cells to platinum by targeting multiple DNA damage response-related genes and thereby determine the prognosis of OC [34]. In addition, miR-211 could sponge lncRNA MALAT1 to suppress tumor growth and progression through inhibiting PHF19 in OC [35]. We could infer that FAM83H-AS1 maybe also play a key role in OC based on above results.

Many evidences taken together suggested that OC patients could potentially benefit from immunotherapy [36]. Identifying immune-related targets could provide assistance for immunotherapy of OC patients. As far as we know, the immune function of FAM83H-AS1 had not been revealed in OC. In our study, we also explore the relationships between immune and FAM83H-AS1. The expression level of FAM83H-AS1 was associated with infiltration level of immune cell including macrophage, neutrphil and dendritic cell in OC patients. The result indicated that FAM83H-AS1 had potential to become a immune-related signature in OC. Our method identified novel candidates associated with OC development and prognosis, which require further research and experimental validation.

## Conclusions

The present study clarified the role of lncRNA FAM83H-AS1 in OC patients. It also demonstrated that the associations between immune and FAM83H-AS1 in OC. The analyses are helpful to identify specific biomarker and drug repurposing candidates for OC. In summary, the present study expands the oncogenic lncRNA landscape of OC and reveals that lncRNA FAM83H-AS1 may act as a potential therapy target for OC.

## Materials and methods

### Obtain and procession of public pan-cancer and OC data

We obtained lncRNA, miRNA, gene expression and methylation (level 3) data, as well as clinical data of all cancer types including OC, from The Cancer Genome Atlas (TCGA, Release: 2017-09-08, https://portal.gdc.cancer.gov/). The gene expression data of normal tissues for ovarian was obtained from GTEX portal (https://www.gtexportal.org/home/index.html). Patients in all the cancer types were integrated as pan-cancer patients. The sample numbers in each cancer type were shown in Table S1. To filter gene, miRNA, and lncRNA not expressed across all samples, the items with expression values of 0 in all of the samples were excluded. Any remaining expression values of 0 were set to the minimum value of all samples, and all values were log2-transformed. Lastly, 14,619 (96.70%) lncRNAs were retained for subsequent analysis. Expression of FAM83H-AS1 was dichotomized using median expression as the cutoff to define “high value” at or above the median versus “low value” below the median.

### Construction of lncRNA-miRNA, lncRNA-protein and lncRNA-mRNA ceRNA networks for FAM83H-AS1

In order to describe the functions of FAM83H-AS1, some interacted regulatory networks were constructed. lncRNA-miRNA network was constructed based on experimentally verified associations between miRNAs and lncRNAs were identified in starBase v3.0 (http://starbase.sysu.edu.cn/) [37] and DIANA-LncBase 3.0 (www.microrna.gr/LncBase) [38]. lncRNA-protein data was download from starBase v3.0 and NPInter v2.0 (http://bigdata.ibp.ac.cn/npinter4) [39] supported by AGO CLIP-seq data. In order to build the lncRNA–mRNA ceRNA network, a hypergeometric test was used to evaluate whether the two lncRNAs have a potential ceRNA relationship by considering their shared interactive miRNAs. *P* < .05 was regarded as statistically significant. All the networks were constructed by Cytoscape 3.3.0 (http://www.cytoscape.org/).

### Obtain of DNA methylation profile of OC

DNA methylation profile was measured experimentally using the Illumina Infinium HumanMethylation450 platform. Beta values were derived at the Johns Hopkins University and University of Southern California TCGA genome characterization center (TCGA, Release: 2017-09-08, https://portal.gdc.cancer.gov/). DNA methylation values, described as beta values, are recorded for each array probe in each sample via BeadStudio software. DNA methylation beta values are continuous variables between 0 and 1, representing the ratio of the intensity of the methylated bead type to the combined locus intensity. Thus higher beta values represent higher level of DNA methylation and lower beta values represent lower level of DNA methylation. Genomic loacations of methylation site were also obtained from TCGA.

### Differential expressed and co-expressed analyses

T- test was used to calculate the differential expression of all genes and lncRNAs between cancer and normal control samples. *P* < .05 was considered as significant differential expressed genes and lncRNA. Pearson’s correlation coefficients (PCCs) were calculated between FAM83H-AS1 and its interacted miRNAs in lncRNA-miRNA network. In addition, co-expression of methylation level and FAM-83H-AS1 expression was also calculated. *P <* .05 was considered as significant co-expressed interactions.

### Survival analysis for the FAM83H-AS1 in pan-cancer and OC

The patients were divided into two groups based on median value of FAM83H-AS1 expression. Kaplan–Meier method and log-rank test were used to evaluate the survival difference in patients with high and low expression of FER1L4. *P <* .05 was regarded as statistically significant.

### Tumor-immune infiltrating cells associated with FAM83H-AS1 in OC

The associations between all tumor-immune infiltrating cells and the FAM83H-AS1 were analyzed via the Tumor Immune Estimation Resource (TIMER) platform (https://cistrome. shinyapps.io/timer/), a web tool for studying tumor-infiltrating immune cells and their interactions with cancer cells [40]. B-cells, CD4 + T-cells, CD8 + T-cells, dendritic cells, macrophages and neutrophils were included in the correlated analyses.

### Patients and tissue samples

OC patients with a histological diagnosis who had undergone surgical resection and had not received chemotherapy or radiotherapy were extracted in our study. Lastly, eight OC tissues and corresponding adjacent normal ovarian tissues were obtained from The Third Affiliated Hospital of Harbin Medical University. The tissue samples were frozen in liquid nitrogen and stored at − 80 °C until experiment. The pathological diagnosis were confirmed by three independent senior pathologists. The study was approved by the Research Ethics Committee of The Third Affiliated Hospital of Harbin Medical University. All patients received written informed consent and disposed of specimens in accordance with accepted ethical standards. The clinicopathological features of all patients are indicated in Table 1.

**Table 1:** The clinicopathological features of all patients

No.	Age (year)	Tumor size (cm)	Lymph node metastasis	Stage	Pathological type	Treatment	ER positive cells (%)	PR positive cells (%)	WT-1	P53	KI67 (%)	MDR
1	53	R:13x13x10	No	IA	Advanced serous adenocarcinoma of right ovary	No	70	< 5%	+	3+	70	+
2	48	L:4.5 × 3.5 × 2.0,R:7.5 × 4.5 × 3.0	No	IIB	Advanced serous adenocarcinoma of bilateral ovary	No	90	40	–	3+	60	–
3	43	L:7x6x5,R:8x6x5	Yes	IIB	Advanced serous adenocarcinoma of bilateral ovary	No	70	30	+	2+	70	–
4	56	L:8x7x7	Yes	IIC	Advanced serous adenocarcinoma of left ovary	No	90	5	+	3+	80	–
5	56	L:8x7x6	No	IA	Advanced serous adenocarcinoma of left ovary	No	70	–	+	3+	–	–
6	53	L:20x16x8	No	IC	Advanced serous adenocarcinoma of left ovary	No	50	10	+	+	30	–

*R* right ovary, *L* left ovary

### Quantitative real-time reverse transcription PCR (qRT-PCR)

Total RNA was extracted from fresh frozen samples and cells using Trizol Reagent (Invitrogen, Carlsbad, CA, USA) according to the manufacturer’s instructions. Total RNA (2 μg) was reverse-transcribed into cDNA using Transcriptor First Strand cDNA Synthesis Kit (Roche, Vilvoord, Brussel, Belgium). The relative levels of EGOT to glyceraldehyde 3-phosphate dehydrogenase (GAPDH) control transcripts were determined by qPCR using the ABI 7500 Fast Real-Time PCR System (Invitrogen). The primer sequences were as follows. FAM83H-AS1: forward 5′-ACTACAGGCACCCACCACCAC-3′, reverse 5′-TGAGACGGGCGGGATCACAAGG-3′; GAPDH: forward 5′-ACCACAGTCCATGCCATCAC-3′, reverse 5′-TCCACCCTGTTGCTGTA-3′. The qRT-PCR amplification was performed in triplicate reactions starting at 95 °C for 10 min, followed by 40 cycles at 95 °C for 10 s, and 60 °C for 60 s. Quantitative normalization of EGOT cDNA was performed in each sample using GAPDH expression as an internal control. The relative level of EGOT transcripts to control GAPDH was determined by the 2 − ΔΔCT method. Each sample was examined in triplicate.

## Supplementary Information

**Additional file 1: Table S1**. The sample numbers in each cancer type.

## Data Availability

The data that support the findings of this study are available from the corresponding author upon reasonable request.
